# Mediterranean Built Environment and Precipitation as Modulator Factors on Physical Activity in Obese Mid-Age and Old-Age Adults with Metabolic Syndrome: Cross-Sectional Study

**DOI:** 10.3390/ijerph16050854

**Published:** 2019-03-08

**Authors:** Antoni Colom, Maurici Ruiz, Julia Wärnberg, Montserrat Compa, Josep Muncunill, Francisco Javier Barón-López, Juan Carlos Benavente-Marín, Elena Cabeza, Marga Morey, Montserrat Fitó, Jordi Salas-Salvadó, Dora Romaguera

**Affiliations:** 1 Instituto de Investigación Sanitaria Illes Balears (IdISBa), University Hospital Son Espases, Palma de 07120 Mallorca, Spain; josep.muncunill@ssib.es (J.M.); margarita.moreyservera@ssib.es (M.M.); 2 CIBER Fisiopatología de la Obesidad y Nutrición (CIBEROBN), Instituto de Salud Carlos III, 28029 Madrid, Spain; jwarnberg@uma.es (J.W.); baron@uma.es (F.J.B.-L.); MFito@imim.es (M.F.); jordi.salas@urv.cat (J.S.-S.); 3 Servicio de SIG y Teledetección, Vicerectorat d’Innovació i Transferència, Universitat de les Illes Balears, 07120 Palma de Mallorca, Spain; maurici.ruiz@uib.es; 4 Departamento de Enfermería, Facultad de Ciencias de la Salud, Universidad de Málaga—Instituto de Investigación en Biomedicina (IBIMA), 29071 Málaga, Spain; 5 Centro Oceanográfico de Baleares, Instituto Español de Oceanografía, 07015 Palma, Spain; montserratcompa@gmail.com; 6 Departamento de Salud Pública, Facultad de Medicina, Universidad de Málaga—Instituto de Investigación en Biomedicina (IBIMA), 29010 Málaga, Spain; jc.benaventemarin@uma.es; 7 Grup d’investigació en Salut Pública de les Illes Balears (GISPIB)—IdiSBa, Servei de promoció de la salut, DG Salut Pública i Participació, Conselleria de Salut, 07010 Palma, Spain; ecabeza@dgsanita.caib.es; 8 Unit of Cardiovascular Risk and Nutrition, Institut Hospital del Mar de Investigaciones Médicas Municipal d’Investigació Mèdica (IMIM), 08003 Barcelona, Spain; 9 Hospital Universitari Sant Joan de Reus, Human Nutrition Unit, IISPV, Department of Biochemistry and Biotechnology, Rovira i Virgili University, 43204 Reus, Spain

**Keywords:** physical activity, accelerometer, leisure, walking, built environment, public open space, GIS, weather, elderly, senior adults, PREDIMED-Plus trial

## Abstract

When promoting physical activity (PA) participation, it is important to consider the plausible environmental determinants that may affect this practice. The impact of objectively-measured public open spaces (POS) and walk-friendly routes on objectively-measured and self-reported PA was explored alongside the influence of rainy conditions on this association, in a Mediterranean sample of overweight or obese senior adults with metabolic syndrome. Cross-sectional analyses were undertaken on 218 PREDIMED-Plus trial participants aged 55–75 years, from the city of Palma, in Mallorca (Spain). Indicators of access to POS and walk-friendly routes were assessed in a 1.0 and 0.5 km sausage network walkable buffers around each participant’s residence using geographic information systems. Mean daily minutes of self-reported leisure-time brisk walking, and accelerometer objectively-measured moderate-to-vigorous PA in bouts of at least 10 min (OM-MVPA) were measured. To investigate the association between access to POS and walk-friendly routes with PA, generalized additive models with a Gaussian link function were used. Interaction of rainy conditions with the association between access to POS and walk-friendly routes with OM-MVPA was also examined. Better access to POS was not statistically significantly associated with self-reported leisure-time brisk walking or OM-MVPA. A positive significant association was observed only between distance of walk-friendly routes contained or intersected by buffer and OM-MVPA, and was solely evident on non-rainy days. In this elderly Mediterranean population, only access to walk-friendly routes had an influence on accelerometer-measured PA. Rainy conditions during the accelerometer wear period did appear to modify this association.

## 1. Introduction

Physical inactivity is a global pandemic: in 2016 more than one in four adults (27.5% or 1.4 billion people) did not meet physical activity recommendations; Spain was slightly below the global average with a physical inactivity prevalence in 2016 of 26.8% (22.9% men and 30.5% women) [1]. Physical inactivity seems to have similar harmful consequences to the established risk factors of smoking and obesity [2]. The association of physical inactivity with major non-communicable diseases makes it a responsible economic burden for health-care systems, having an estimated cost for European countries of 15.5 billion dollars and 2.3 billion dollars in Spain [3]. Regular physical activity (PA) on the other hand, has shown to reduce the risk of cardiovascular disease, hypertension and diabetes and can help maintain a healthy weight [2]. This being so, it has been highlighted that older adults are the least physically active age group [4], which is especially concerning since the proportion and number of older people is increasing dramatically [5]. Additionally, more than half of all older people in high-income countries are affected by multimorbidity [6]. Specifically, in Spain, one-half to two-thirds of Spanish adults older than 65 have two or more chronic conditions [7]. All this makes healthy ageing an emerging key policy issue globally [8].

A wide range of environmental factors have been attributed to facilitating PA as a means of transportation and recreation. On the one hand, urban built environments, like walk-friendly routes, and public open spaces (POS), such as parks, beaches, and sports facilities, are plausible determinants of PA carried out by older adults, but some controversy exists between studies [9,10]. On the other hand, only a few studies, in Northern European populations, have explored the impact of weather conditions on PA practiced in older adults [11,12,13], leaving out the Mediterranean countries which have one of the highest prevalences of physical inactivity in the European Region [14].

A previous study has explored the cross-sectional relationship between access to POS and total self-reported leisure time PA in elderly participants at high cardiovascular risk from a previous trial PREDIMED-Baleares [15]. In this previous study, no association between access to POS and PA was observed; however, like other previous studies, there were important limitations. Mainly, the total PA was only self-reported, evaluated from a validated questionnaire, but no objectively-measured data were available. In addition, leisure PA data used did not allow types of activity to be differentiated (i.e., leisure-time walking), making it context-free. Moreover, it was not possible to evaluate the influence of weather conditions, given that the questionnaire reported frequency and duration of total PA performed during a representative month.

In this study, we aimed to explore the joint interaction of both factors: POS and walk-friendly routes with weather conditions and their effect on self-reported and objectively measured PA in an elderly local population in the Western Mediterranean Sea. The cross-sectional association of access to POS and walk-friendly routes using self-reported and objectively-measured PA, with the interaction of rainy conditions was investigated with this association in older adults participating in the PREDIMED-Plus-Baleares trial.

## 2. Materials and Methods

### 2.1. Study Population

The PREDIMED-Plus trial is an ongoing six-year multi-center, randomized, parallel-group trial, designed to evaluate a lifestyle strategy for the primary prevention of cardiovascular mortality in 6874 senior adults. The PREDIMED-Plus study protocol details have been described elsewhere [16] (the protocol is available at http://predimedplus.com/ and the trial was registered at the International Standard Randomized Controlled Trial http://www.isrctn.com/ISRCTN89898870). 

Participants were community-dwelling adults (aged 55–75 in men; 60–75 in women) with BMI ≥ 27 and <40 kg/m^2^, enrolled from primary care facilities in Spain between the 5th of September in 2013 and the 31st of October in 2016 who had at least 3 or more metabolic individual components of the syndrome [17]. The lifestyle intervention consists of an energy-restricted traditional Mediterranean diet (MedDiet), PA promotion, and behavioral support, in comparison to a usual care intervention with only an energy-unrestricted MedDiet (control group). 

This is a cross-sectional analysis of baseline data (dataset extracted 1/15/18) of 335 participants enrolled from primary care facilities dependent on University Hospital Son Espases, one of the 23 recruitment centers participating in the PREDIMED-Plus trial. The PREDIMED-Plus study protocol and ethical approval was granted by the Committee of Research Ethics of the Balearic Islands (CEI-IB) review boards and all participants provided written informed consent. The ethics approval number given to this study was IB-2242/14-PI. For this geographic sub-study within the PREDIMED-Plus project, following the regulations of the CEI-IB, an amendment was requested and favorably evaluated.

Participants who reported living outside the city limits of Palma de Mallorca (*n* = 16), those with no accelerometer data (wore no accelerometer at all; *n* = 100), and those with fewer than four days of valid accelerometer data (*n* = 1), were excluded from the analyses, leaving a final sample size of 218 participants.

### 2.2. Neighborhood Exposure to Walk-Friendly Routes and Public Open Spaces (POS) 

**Figure 1:**
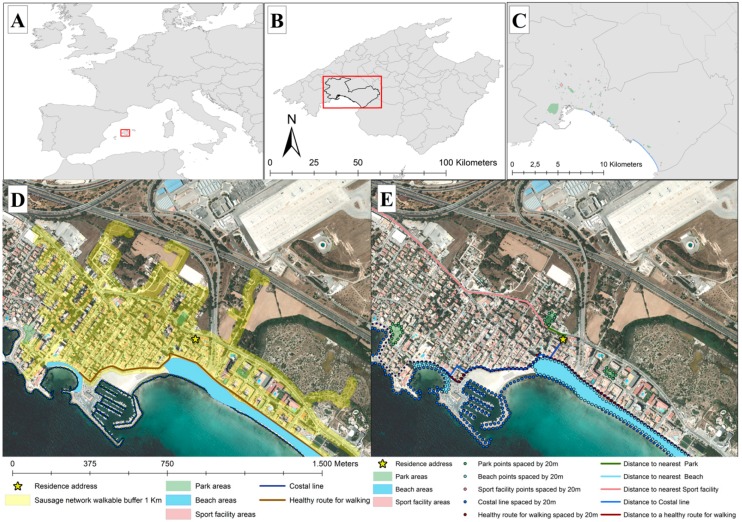
Study area of Palma, Mallorca (**a–c**). Example of neighborhood exposure to public open spaces (POS) with a 1 km sausage network walkable street buffer around the residence address represented by the yellow area (**d**), and the point boundaries of the built environments (**e**).

POS were evaluated using the geographic area around each participant’s home. The location of each participant’s residence, reported at baseline, was geocoded using the web processing service provided by Carto Ciudad, a project run by the National Geographic Institute of Spain, which is freely available for download (www.cartociudad.es). Using the software ArcGIS 10.5.1 (ESRI, Redlands, CA, USA), for each participant’s residence location, a sausage network buffer methodology was applied. The full methodology has been described elsewhere [18], briefly: using only the walking street network, ignoring routes restricted to pedestrians such as freeways, the area within 1 km and 0.5 km walkable street distance of each participant’s residence location was obtained, then, 30 m of each road length were buffered; as a result, it was possible to obtain the space where each participant could move along the street walkable network and a radius at an amount of 30 m either side of the street network (Figure 1). Next, using the tabulate intersection tool from ArcGIS 10.5.1 software, different POS were quantified within or partially within each participant’s residence sausage network walkable buffer. Details on the steps to build this buffer are included in a protocol available online [19]. The capacity of these buffers to predict the results of physical activity have been described similar that other buffers (circular buffer or detailed buffer), although sausage buffers have a substantial advantage over others buffers by quantifying the built environment. Sausage buffers only count the features that are accessible from the road network regardless of street network connectivity [18,20].

In order to determine the distance to the closest POS, walk-friendly routes in the absence of POS and walk-friendly route entry points, the boundaries of each polygon or line were transformed into points spaced 20 m from each other. Since the shape and the size of the resources are heterogenous, by converting the polygons and lines into predefined spaced points, we were able to reduce the measurement error [21] (Figure 1). The next step was to identify the closest distance to each of the POS boundaries and the nearest walk-friendly routes for each participants residence. This was achieved by calculating the walking street network distance using the Origin-Destination Cost Matrix function from ArcGIS 10.5.1.

Public Open Spaces (POS)—For the POS in this study, we calculated the proximity, count and total area, all of which are commonly used in PA and health research. Our POS were generated for three different data sources for sports facilities, beaches and parks. Sports facilities data was generated from the Municipal Sports Institute of the City of Palma. Only public sports facilities owned and maintained by the City of Palma were considered. A full list is detailed in Colom et al., 2018 [15]. Since our target area was for public areas only, private sports facilities, such as golf courses, private gyms, and private courts, were not included in this study. In addition, the City of Palma is in close proximity to the coastline and has a considerable number of beaches with lifeguard services during the summer season; all beaches with this service were included. Finally, the dataset containing all of the parks was acquired from the Department of Infrastructure and Accessibility from the Palm City Council.

The number of sports facilities and parks, and the area of parks within or partially within the 1000 and 500 m sausage network walkable buffer were computed separately, as well as the sum of the number and area of all types of POS. The number of beaches and the area of sports facilities and beaches were excluded due to the high number of zeros.

Distance to the nearest POS from each participant’s residence were considered separately (sports facilities, beaches and parks). Distance to any POS was not considered exposure because for 85.3% of the sample, the nearest POS was a park. Proximity of each participant to the coast as a potential resource for doing PA was included, since the city of Palma is a coastal city [22]. 

In addition, distance inside the buffer and proximity to walk-friendly routes for healthy walking and coast line were also considered, as they might directly or indirectly influence active travel [23]. Walk-friendly routes were part of a project called “Rutas Saludables” launched by local government health policies. For the design of these routes, the coordinators had the collaboration of neighborhood associations and citizens in the neighborhoods involved, as well as municipal managers, and they were promoted at the primary health care centers in the city.

### 2.3. Outcome Measure: Physical Activity

For this analysis both objectively-measured PA and self-reported PA, assessed at baseline, were evaluated. 

Self-reported leisure-time brisk walking—Participants completed the Girona Heart Registry (REGICOR) Short Physical Activity Questionnaire [24], a validated short version of the validated Spanish Minnesota leisure-time PA questionnaire (MLTPAQ) [25,26]. The REGICOR questionnaire collects information on walking, prior analysis of the main mode of physically active transportation [24], and includes a specific question to collect information about leisure-time brisk walking, frequency (number of days), and duration (min/day) performed during a representative month. Time per day spent on leisure-time brisk walking (accumulated minutes/day) was computed as the sum of frequency multiplied by duration of the activity, divided by 30. Dichotomous outcome measures were computed to represent the recommendations defined by the World Health Organization any ≥150 min per week of moderate-to-vigorous physical activity such as leisure-time brisk walking [27]. 

Objectively-measured moderate-to-vigorous physical activity in bouts of at least 10 minutes (OM-MVPA)—Participants at the baseline visit were asked to wear a wrist-worn triaxial accelerometer (GENEActiv, ActivInsights Ltd, Kimbolton, UK), on their non-dominant wrist nonstop for seven consecutive 24-h days. The accelerometer was sampled at 40 Hz with a ± 8 *g* dynamic range, and data were stored in gravity (*g*) units (1 *g* = 9.81 m/s^2^). Accelerometer data were processed and scored using R version 3.3.3 (R Core Team, Vienna, Austria) and R-package GGIR (version 1.2-5), available on CRAN (https://cran.r-project.org) and managed on servers at the University of Malaga, one of the 23 recruitment centers participating in the PREDIMED-Plus trial. For scoring PA, a ‘valid day’ was defined as ≤2 h non-wear per day. Only participants with four or more valid days were included in analyses [28]. These methods were consistent with other authors’ recommendations [29]. The maximum time was nine consecutive 24-h days (both daytime and nighttime). Objectively-measured moderate-to-vigorous physical activity was calculated as accumulated minutes/day in intensities greater than 100 milligravity [30], and bouts of at least 10 min of moderate-to-vigorous physical activity. Dichotomous outcome measures were computed to represent World Health Organization recommendations of ≥150 min of moderate-to-vigorous physical activity a week [27]. 

### 2.4. Covariate Assessment

Covariable information on sex, age, education level (primary school or less; secondary school or higher), and self-rated health were collected at the baseline using a self-reported questionnaire. Self-rated health was measured by the question, “How would you rate your general health?” from an adapted version of previously published questionnaires on health-related quality of life that had been validated for the Spanish population [31]. Participants reporting excellent, very good and good health were categorized into one group and those reporting fair and poor health in the other group.

### 2.5. Weather Assessment

**Figure 2:**
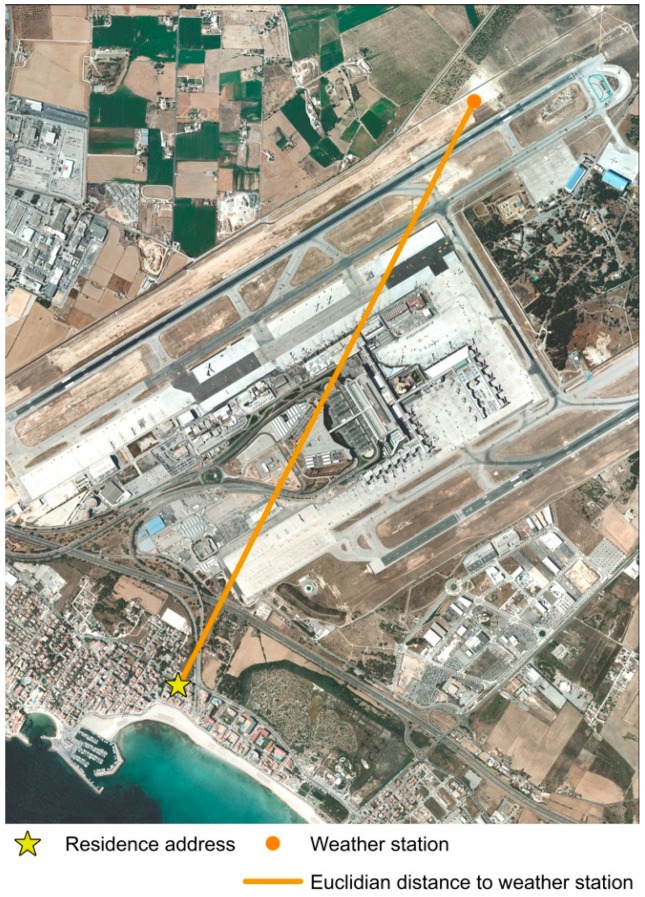
Example of closest Euclidian distance from participants’ resident weather station.

Using the software ArcGIS 10.5.1, for each participant’s residence location, Euclidian distance or distance “as the crow-flies” was applied in order to obtain the closest weather station from each participant resident location previously geocoded (Figure 2). Using the application programming interface provided by the OpenData service of the Agencia Estatal de Meteorología (AEMET) we collected the accumulated precipitation (mm) for each day during accelerometer wearing period. As we do not have access to each day physical activity accelerometer data, mean measurements of precipitation (mm) data was calculated for each participant during accelerometer wearing period. Since a large number of days had no precipitation (i.e., 0 mm), the variable was dichotomized into two groups: without rain and with rain. 

### 2.6. Statistical Analysis

A descriptive analysis for the outcome variables (min/day) of self-reported leisure-time brisk walking and OM-MVPA was performed by comparing participant demographic characteristics and the precipitation variable. A one-way analysis of variance (ANOVA) was used to evaluate differences in baseline self-reported leisure-time brisk walking and OM-MVPA based on demographics.

Associations of objectively-measured POS variables with self-reported leisure-time brisk walking and OM-MVPA were estimated using generalized additive mixed models (GAMMs) [32]. GAMMs can assume data with various distributional assumptions, and also cover mixed-effects models, accounting for dependency in error terms due to clustering (in our case, participants enrolled from selected primary care facilities), and estimate complex dose-response by handling non-linear, linear, and non-monotonic relationships [33]. Preliminary analyses indicated that GAMMs with Gaussian variance and identity link functions would be most appropriate for the continuous outcomes: minutes/day of self-reported leisure-time brisk walking, and minutes/day of OM-MVPA 10-min bouts. Regression coefficient estimates of these GAMMs represent the change in outcomes per increment 1 km, 1 km^2^, or count in exposure to POS. GAMMs with binomial variance and logit link functions were the most appropriate for the dichotomized outcomes: any ≥150 min per week of self-reported leisure-time brisk walking and any ≥150 min per week of OM-MVPA 10-min bouts. Antilogarithms of the regression coefficient estimates of these GAMMs represent odds ratios. 

The curvilinearity relationships were assessed with thin-plate spline smooth terms [32,33] adjusted for sex, age (≤65 years vs. >65 years), education level (primary or less vs. more than primary education), and self-rated health (excellent/very good/good vs. fair/poor). For all GAMMs, random intercepts were specified to account for clustering effects at the administrative unit level, as participants were enrolled from selected primary care facilities. Analyses of a curvilinear relationship were conducted for each model observing the Akaike Information Criterion (AIC), differences ≥10 caused the smooth terms of the model to be removed and replaced by a simpler linear term [34]. 

Further, interaction of weather conditions specifically rainy conditions, with the association of objectively-measured POS and walk-friendly route variables with OM-MVPA was also examined. Significance of interactions was assessed by adding cross-product terms between our exposure of interest and rainy conditions (classified in two categories: rainy or non-rainy periods). Then for the walk-friendly route measures that presented significant interactions, stratified analyses were performed, by examining the association of these POS measures and OM-MVPA in rainy periods and non-rainy periods, separately.

All analyses were conducted in R version 3.3.3 (R Development Core Team, Vienna, Austria) using ‘stats’ [35], ‘mgcv’ [33], and ‘gamm4’ [36] packages. 

## 3. Results

### 3.1. Descriptive Statistics

Table 1 shows the descriptive characteristics of 218 participants with at least four valid days of OM-MVPA data. The mean age was 65.2 years with all participants ranging from 55 to 75 years. Almost 40% of participants had reached secondary school or higher and around 70% of participants reported excellent/very good/good health. Taking into account self-reported leisure-time brisk walking, 61.5% of the population (44.8% men and 55.2% women) and according to OM-MVPA, 72.0% of the population (40.8% men and 59.2% women) did not meet the minimum recommended level of WHO Physical Activity Guidelines (i.e., at least 150 min/week of moderate-to-vigorous physical activity) [27] (Table 1). On average, participants accumulated about eight (±1.3) valid days of accelerometer wear (data not shown in Tables). In our study, poorer self-rated health was associated with a lower level of PA (Table 1). Women showed significantly lower OM-MVPA than men. Precipitation was not significantly associated with either self-reported or objectively-measured PA in this sample.

**Figure 3:**
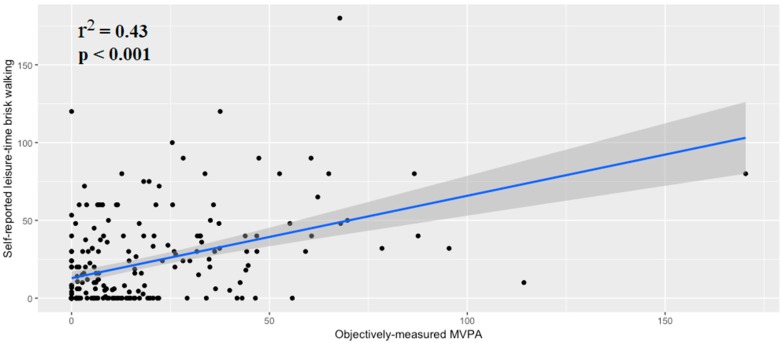
Correlations between self-reported leisure-time brisk walking and OM-MVPA. Abbreviations: *p*, *p*-value; *r*^2^, coefficient of determination; association between variables were evaluated using the Pearson test. OM-MVPA: objectively-measured moderate-to-vigorous PA in bouts of at least 10 min.

Figure 3 shows the correlations between self-reported leisure-time brisk walking and OM-MVPA, assessed at baseline. Notably, self-reported leisure-time brisk walking and OM-MVPA were significantly and strongly correlated with each other (Figure 3). When concordance of the classification of meeting or not meeting the WHO recommendation on PA was assessed using self-reported and objectively-measured data, an intraclass correlation coefficient of 0.41 was observed, indicating a fair correlation. 

Total number of POS within or partially within the sausage network walkable buffer was 10.03 (±5.91) with a mean area of 0.17 (±0.25) km^2^ (Table 2). The furthest distance to a POS was distance to the nearest beach with a mean of 3.9 km, followed by distance to the coast of just under 3 km; conversely there was a considerably short distance to the nearest park of under 0.3 km. Our population had an average of 4.24 (±3.15) km of walk-friendly routes, but only 0.04 (±0.12) km of coastline contained or intersected within the 1000 m sausage network walkable buffer. The largest number of POS within or partially within the sausage network walkable buffer were parks with a mean number of 8.5 (±5.09) and a mean area of 0.16 (±0.25) km^2^. Supplementary Table S1 shows the descriptive statistics of objectively-assessed POS for a 500 m sausage network walkable street buffer.

**Figure 4:**
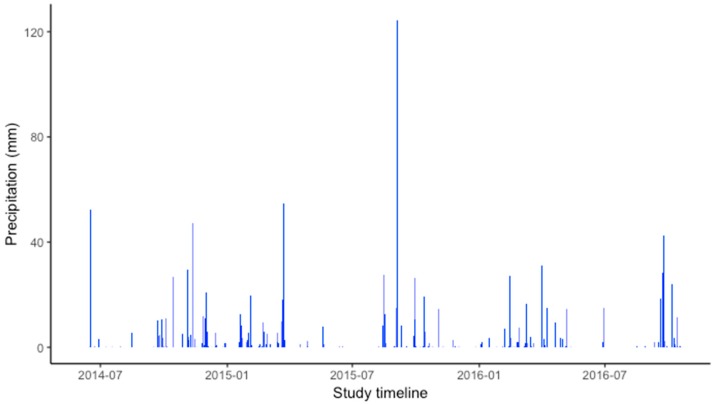
Mean daily precipitation (mm) during the study from participants’ closest resident weather station.

Precipitation throughout the study is reflected in Figure 4; during the accelerometer wearing period daily precipitation ranged from 0 to 17.4 mm, whereas throughout the period there was an accumulated average of 0.9 mm and 53% completely dry days with no precipitation.

### 3.2. Associations of POS with Self-Reported Leisure-Time Brisk Walking and OM-MVPA

Table 3 and Table 4 show the association between distance to POS or walk-friendly route, sums of areas, and counts or sums of distances within each sausage network walkable buffer of 1 km and self-reported leisure-time brisk walking and OM-MVPA respectively. Both self-reported leisure-time brisk walking and OM-MVPA were adjusted by individual-level covariates. 

Overall, no statistically significant associations were observed between POS or walk-friendly routes and self-reported leisure-time brisk walking or OM-MVPA. Only two borderline significant associations were observed: one negative borderline association between area of parks contained or intersected by the buffer and self-reported leisure-time brisk walking (*p* = 0.077); and another positive borderline association between distance of walk-friendly routes contained or intersected by buffer and OM-MVPA (*p* = 0.052). No associations were found between any POS or walk-friendly routes and meeting World Health Organization recommendations.

Overall, no statistically significant associations were observed between POS or walk-friendly routes in the 500 m buffer and self-reported leisure-time brisk walking or OM-MVPA (Supplementary Tables S2 and S3).

The interaction between each POS and walk-friendly routes and rain was tested during the accelerometer-wearing period on OM-MVPA. The only significant interaction was observed between the distance of walk-friendly routes contained or intersected by the 1000 m buffer and OM-MVPA (interaction: *p* = 0.028) and a borderline significant interaction between distance of walk-friendly routes contained or intersected by the buffer and engaging in ≥150 min/week OM-MVPA (interaction: *p* = 0.071) (Supplementary Table S4). Overall, no statistically significant interactions were observed between POS in the 500 m buffer and OM-MVPA (Supplementary Table S5).

Then the association between distance of walk-friendly routes contained or intersected by the sausage buffer and OM-MVPA was analyzed in non-rainy periods and rainy periods, separately (stratified analyses). For non-rainy periods a positive significant association was found (β = 1.812; 95% CI = 0.375–3.249; *p* = 0.015). Increments of 1 km of walk-friendly routes contained or intersected by the 1 km sausage network walkable buffer, were associated with an increase of 1.8·min/day of OM-MVPA; however, during rainy periods, no significant association was found (β = −0.063; 95% CI =−1.174–1.047; *p* = 0.911) between walk-friendly routes and OM-MVPA. On the whole, for rainy periods no statistically significant interactions were observed between any POS and models of meeting World Health Organization recommendations.

## 4. Discussion

### 4.1. Main Findings

This study investigated the association between access to POS as well as walk-friendly routes on self-reported and objectively-measured PA, and the interaction of rainy conditions with this association in older adults participating in the PREDIMED-Plus-Baleares. Overall findings revealed that increments in km of walk-friendly routes contained or intersected by 1 km sausage network walkable buffer were associated with an increase in 10-minute bouts of OM-MVPA, although this association was only evident on non-rainy days.

### 4.2. Built Environment, Weather, and Physical Activity in Older Adults

The findings of this study show that urban built environments, such as POS, are not associated with either self-reported or objectively-measured PA in older adults. In addition, we found no association between any POS or walk-friendly route and compliance with World Health Organization recommendations. 

Older adults are the least physically active age group [4]. Despite their greater free time due to the release of their work life and more opportunity to practice leisure time PA, it does not compensate the physical activity that they had when they worked [37,38]. Individual characteristics, such as poorer health status, has been reported to lower leisure time PA in older adults [39,40]. Additionally, environmental characteristics, such as weather conditions especially rainfall, have been reported as an important barrier for older adults get to reach the recommendations in leisure time PA [41]. Socio-ecological models postulate that PA in older adults is influenced by factors at multiple levels (individual, psychosocial and environmental characteristics) and also by the interactions between these factors at multiple levels [42] In this context, built environments (i.e., POS) offer an important resource for public health [43]. 

Our results are in line with our previous results based on another population that showed, in the same city, non-significant relationships between objectively assessed POS accessibility and only self-reported total leisure time PA [15]. On the other hand, there is evidence that POS are positive associated to total PA in older adults, however, only perceived access to POS was positively associated with total PA; conversely, objectively assessed POS, as in our studies, were not found to be associated to total PA [10]. Moreover, no relationship was found between POS and objectively-measured PA [10]. A recently published systematic review and meta-analysis in older adults found that POS was positively related to total leisure time PA, but in contrast no association was found between POS accessibility and leisure-time walking [9]. We found that a novel urban built environment, POS, such as km of walk-friendly healthy routes promoted by the local government, contained or intersected by a 1 km sausage network walkable buffer was associated with objectively-measured PA. Furthermore, this increment almost doubled when only non-rainy periods were included. Although we were unable to differentiate whether these walk-friendly healthy routes correlated with other common urban built environment measures commonly used in PA and health research such as residential density, public transport stop access, road intersection density, land use mix, and or walkability index, we suspect these conditions might strongly affect local government health policies like the construction of these walk-friendly routes. Moreover, these urban built environment measures demonstrate significant relationships between POS accessibility and leisure-time walking [9].

Despite the fact that only walk-friendly routes were observed to have a significant interaction with rain during the accelerometer-wearing period on OM-MVPA, it seems plausible to consider that rainy conditions might reduce outdoor activity in older adults. Qualitative research findings indicate that concerns over safety, fear of falling and injury are potential barriers to perform outdoor activity in older adults [44]. Although this was beyond the scope of this study, it might be possible that older individuals have such safety concerns, among others, when walking on rainy days. Our findings may also be explained by the characteristics of the Mediterranean climate in our study area with fewer rainy periods compared to some other regions.

### 4.3. Strengths and Limitations

The strengths of the study include the fact that it was based on a baseline data of an ongoing Spanish randomized controlled trial, including senior overweight and obese adults with metabolic syndrome with self-reported and objective measures of PA over seven days and POS data. Interaction of rainy conditions was also evaluated through. Therefore, this study advances in the exploration of the environment and physical activity in seniors including the rain as a plausible effect modifier. Further, weather measurements were taken from the closest weather station for each participants’ resident address. We studied self-reported leisure-time brisk walking and 10-minute bouts OM-MVPA in order to be more context-specific; as opposed to context-free measures of PA such as total amount of PA. For our population, both PA measures (self-reported leisure-time brisk walking and OM-MVPA) were significantly correlated with each other. A wide range of POS were evaluated, including access to beach, coast, and walk-friendly routes, variables that are relevant to our population and may influence leisure-time walking [23]. For the evaluation of access to POS, a sausage network walkable street buffer was used rather than walkable street buffers or a radial buffer, in order to minimize measurement errors [20].

In terms of limitations, the design of this study it is cross-sectional. Therefore, although we can identify factors that might be targeted to improve PA in future studies, we cannot infer causality or establish that the exposures preceded the outcomes. Additionally, there are other variables that could modify the association between POS and PA, such as age, sex, self-rated health or educational level. However, the small sample size precluded us to perform any of those stratified analyses and other sensitivity analyses. Although pluviometry during seasonality changed throughout the study period, the general climate of this area is mild with less extreme rainfall conditions compared to some regions of Spain or other countries.

Furthermore, in this study, the study population was enrolled from medium-sized primary care facilities in the city of Palma de Mallorca, an island located in the Mediterranean Region of Spain. The population under study is a homogeneous sample of aged participants with obesity and metabolic syndrome, and overall low PA levels. 

## 5. Conclusions

When promoting PA, it is important to consider the plausible environmental determinants that may affect this practice. In this elderly population living in a Mediterranean city, km of walk-friendly routes contained or intersected by a 1 km sausage network walkable buffer influenced accelerometer-measured moderate-to-vigorous physical activity 10-min bouts and rainy conditions during the accelerometer wear period did appear to be an important factor related to active ageing. Future work should focus on a larger sample size and greater heterogeneity in built environment access. Also, integrating environmental data (i.e., built environment and weather conditions) on a longitudinal physical activity intervention trial may help to evaluate how these factors influence the adherence to the intervention among older adults. The PREDIMED-Plus study as a multi-centre randomized trial presents an excellent opportunity for future urban environmental studies.

## Figures and Tables

**Table 1:** Average minutes/day of self-reported leisure-time brisk walking and OM-MVPA by study population demographic characteristics.

Individual/Demographic		Time Physical Activity (min/day)			
	*n* (%)	Self-Reported Leisure-Time Brisk Walking	*p*	Objectively-Measured MVPA 10-min Bouts	*p*
All	218 (100)	22.0 (28.4)		17.3 (23.0)	
Sex			0.599		<0.001
Men	106 (48.6)	23.0 (26.9)		23.0 (28.1)	
Women	112 (51.4)	21.0 (29.8)		11.8 (15.1)	
Age (years)			0.879		0.123
>65	102 (46.8)	22.3 (31.0)		14.7 (18.8)	
≤65	116 (53.2)	21.7 (25.9)		19.5 (26.1)	
Educational level			0.238		0.118
Primary school or less	132 (60.6)	20.2 (29.9)		15.3 (19.1)	
Secondary school or higher	86 (39.4)	24.8 (25.7)		20.3 (27.9)	
Self-rated health			0.010		<0.001
Excellent/very good/good	145 (67.1)	24.6 (27.9)		20.2 (25.8)	
Fair/poor	71 (32.9)	14.8 (21.7)		10.5 (13.1)	
Precipitation (mm)			0.731		0.237
Non-rainy period	119	21.4 (28.5)		18.9 (26.4)	
Rainy period	99	22.7 (28.3)		15.2 (18.2)	

Values shown are mean (SD) unless otherwise specified; *p*, *p*-value. The *p*-value is computed from the ANOVA test. OM-MVPA: objectively-measured moderate-to-vigorous PA in bouts of at least 10 min.

**Table 2:** Descriptive statistics of objectively-assessed POS for a 1000 m sausage network walkable street buffer.

Objectively-Assessed POS	Mean (SD)	*n* Zeros (%)
Distance to coast (km)	2.98 (1.71)	0 (0.00)
Distance to walk-friendly route (km)	0.45 (0.58)	0 (0.00)
Distance to nearest sports facility (km)	0.85 (0.66)	0 (0.00)
Distance to nearest beach (km)	3.89 (1.55)	0 (0.00)
Distance to nearest park (km)	0.28 (0.4)	0 (0.00)
Coastline contained or intersected by buffer (km)	0.04 (0.12)	193 (88.53)
Walk-friendly routes contained or intersected by buffer (km)	4.24 (3.15)	22 (10.09)
No. sports facilities contained or intersected by buffer	1.49 (1.34)	66 (30.28)
Area of sports facilities contained or intersected by buffer (km^2^)	0.02 (0.03)	66 (30.28)
No. parks contained or intersected by buffer	8.5 (5.09)	9 (4.13)
Areas of parks contained or intersected by buffer (km^2^)	0.16 (0.25)	9 (4.13)
No. beaches contained or intersected by buffer	0.04 (0.19)	210 (96.33)
Areas of beaches contained or intersected by buffer (km^2^)	0 (0)	210 (96.33)
No. POS contained or intersected by buffer	10.03 (5.91)	9 (4.13)
Areas of POS contained or intersected by buffer (km^2^)	0.17 (0.25)	9 (4.13)

Values shown are mean (SD) and *n* (%) for variables. Abbreviations: SD, Standard deviation; POS, public open spaces; km, Kilometer; km^2^, Square kilometer.

**Table 3:** Summary of associations between objectively-measured access to public open spaces (POS) by distance and a 1000 m sausage network walkable street buffer and self-reported leisure-time brisk walking measure. Results for the following model comparisons are provided: Covariate-adjusted POS GAMMs.

Predictor Variable	^a^ Self-Reported Leisure-Time Brisk Walking (Minutes/Day) (*n* = 216)			^b^ Engaging in ≥ 150 min/week Self-Reported Leisure-Time Brisk Walking (Yes = 83 (38.4%); No = 133 (61.6%))		
	β	95% CI	*p*	OR	95% CI	*p*
Distance to the coast (km)	2.063	−0.005–4.131	0.052	1.095	0.924–1.298	0.294
Healthy routes contained or intersected by buffer (km)	2.374	−3.762–8.51	0.449	0.917	0.558–1.504	0.73
Distance to nearest sports facility (km)	−2.541	−7.836–2.754	0.348	0.96	0.625–1.473	0.85
Distance to nearest beach (km)	0.518	−1.742–2.778	0.654	0.959	0.798–1.152	0.651
Distance to nearest park (km)	3.134	−5.662–11.929	0.486	1.124	0.557–2.267	0.744
Walk-friendly routes contained or intersected by buffer (km)	−0.133	−1.246–0.98	0.815	1.015	0.928–1.11	0.751
No. sports facilities contained or intersected by buffer	0.339	−2.266–2.944	0.799	0.956	0.774–1.18	0.676
No. parks contained or intersected by buffer	−0.215	−0.905–0.475	0.542	0.995	0.941–1.052	0.867
Areas of parks contained or intersected by buffer (km^2^)	−12.685	−26.678–1.309	0.077	0.54	0.163–1.793	0.315
No. POS contained or intersected by buffer	−0.123	−0.716–0.469	0.684	0.995	0.949–1.044	0.851
Areas of POS contained or intersected by buffer (km^2^)	−11.579	−25.545–2.388	0.106	0.58	0.178–1.891	0.368

Abbreviations: β, non-standardized coefficient; OR, odds ratio; 95%CI, confidence interval; *p*, *p*-value. β indicates change in self-reported leisure-time brisk walking according to minutes per day (min/day) per increment (in 1 km, 1 km^2^, or count) in access to public open spaces (POS). All coefficients were adjusted for individual-level covariate (sex, age, education level, and self-rated health). ^a^ GAMMs with Gaussian variance and identity link functions. ^b^ GAMM with binomial variance and logit link functions.

**Table 4:** Summary of associations between objectively- measured access to public open spaces (POS) by distance and a 1000 m sausage network walkable street buffer and 10-minute bouts OM-MVPA. Results for the following model comparisons are provided: Covariate-adjusted POS GAMMs.

Predictor Variable	^a^ Objectively-Measured MVPA 10 Min Bouts (Minutes/Day) (*n* = 216)			^b^ Engaging in ≥ 150 min/wk Objectively-Measured MVPA 10 Min Bouts(Yes = 60 (27.8%) No = 156 (72.2%))		
	β	95% CI	*p*	OR	95% CI	*p*
Distance to coast (km)	0.106	−1.9–2.113	0.917	1.055	0.876–1.273	0.571
Distance to walk-friendly route (km)	1.088	0.647–1.828	0.752	1.088	0.647–1.828	0.752
Distance to nearest sports facility (km)	−1.137	−6.173–3.898	0.658	1.037	0.651–1.649	0.881
Distance to nearest beach (km)	−1.099	−3.234–1.035	0.314	0.938	0.764–1.152	0.542
Distance to nearest park (km)	−3.56	−12.956–5.835	0.459	0.875	0.404–1.894	0.735
Walk-friendly routes contained or intersected by buffer (km)	0.981	−0.004–1.966	0.052	1.031	0.933–1.139	0.548
No. sports facilities contained or intersected by buffer	0.994	−1.275–3.264	0.391	1.055	0.839–1.327	0.645
No. parks contained or intersected by buffer	0.485	−0.132–1.102	0.125	1.016	0.955–1.082	0.609
Areas of parks contained or intersected by buffer (km^2^)	−3.86	−15.756–8.036	0.525	0.695	0.189–2.558	0.585
No. POS contained or intersected by buffer	0.398	−0.132–0.928	0.143	1.014	0.962–1.069	0.599
Areas of POS contained or intersected by buffer (km^2^)	−3.751	−15.597–8.095	0.536	0.724	0.199–2.632	0.624

Abbreviations: β, non-standardized coefficient; OR, odds ratio; 95% CI, confidence interval; *p*, *p*-value; MVPA, moderate-to-vigorous physical activity in bouts of at least 10 min; GAMMs: generalized additive mixed models. β, indicates change in 10-min bouts objectively-measured-MVPA according to minutes per day (min/day) per increment (in 1 km, 1 km^2^, or count) in access to public open spaces (POS). All coefficients are adjusted for individual-level covariate (sex, age, education level, and self-rated health). ^a^ GAMMs with Gaussian variance and identity link functions. ^b^ GAMM with binomial variance and logit link functions.

## Supplementary Materials

The following are available online at https://www.mdpi.com/1660-4601/16/5/854/s1, Table S1: Descriptive statistics of objectively-assessed POS for a 500 m sausage network walkable street buffer, Table S2: Summary of associations between objectively-measured access to POS for a 500 m sausage network walkable street buffer and self-reported leisure-time brisk walking measure, Table S3: Summary of associations between objectively-measured access to POS for a 500 m sausage network walkable street buffer and 10-min bouts OM-MVPA, Table S4: Summary of the interaction between objectively-measured access to Public Open Spaces (POS) according to distance and a 1000 m sausage network walkable street buffer and 10-min bouts OM-MVPA, Table S5: Summary of the interaction between objectively-measured access to public open spaces (POS) for a 500 m sausage network walkable street buffer and 10-min bouts OM-MVPA.

## Appendix A

PREDIMED-PLUS Investigators, University Hospital Son Espases (HUSE) and Instituto de Investigación Sanitaria Illes Balears (IdISBa).

Fiol M, Moñino M, Konieczna J, Zamanillo R, Galmés AM, Pereira V, Martín MA, Yáñez A, Llobera J, Ripoll J, Prieto R, Grases F, Costa A, Fernández-Palomeque C, Fortuny E, Noris M, Munuera S, Tomás F, Fiol F, Jover A, Janer JM, Vallespir C, Mattei I, Feuerbach N, Del Mar Sureda M, Vega S, Quintana L, Fiol A, Amador M, González S, Coll J, Moyá A, Piqué T, Sanmartín MD, and Peña C.
