# Supplementary material for: Mediterranean Built Environment and Precipitation as Modulator Factors on Physical Activity in Obese Mid-Age and Old-Age Adults with Metabolic Syndrome: Cross-Sectional Study
Source: Int J Environ Res Public Health. 2019 Mar 8;16(5):854. doi: 10.3390/ijerph16050854 (PMC6427354; doi:10.3390/ijerph16050854)
Supplement: Supplementary file 1 [file ijerph-16-00854-s001.pdf]

Supplementary Table S1. Descriptive statistics of objectively-assessed POS for a 500 m sausage network walkable street buffer. Values shown are mean (SD).

**Objectively-Assessed POS**	**Mean (SD)**
Coast line contained or intersected by buffer (km)	0 (0.03)
Healthy routes contained or intersected by buffer (km)	1.11 (1.1)
No. sports facilities contained or intersected by buffer	0.52 (0.73)
Area of sports facilities contained or intersected by buffer (km^2)	0 (0.01)
No. parks contained or intersected by buffer	2.88 (1.88)
Areas of parks contained or intersected by buffer (km^2)	0.08 (0.21)
No. beaches contained or intersected by buffer	0.02 (0.13)
Areas of beaches contained or intersected by buffer (km^2)	0 (0)
No. POS contained or intersected by buffer	3.42 (2.21)
Areas of POS contained or intersected by buffer (km^2)	0.08 (0.21)

Abbreviations: SD, standard deviation; POS, public open spaces; km, Kilometer; km^2, square kilometer.

Supplementary Table S2. Summary of the results associations between objectively-assessed public open spaces (POS) for a 500 m sausage network walkable street buffer and self-reported leisure-time brisk walking measure and 10-minute bouts of OM-MVPA. The results for the following model comparisons are provided: Covariate-adjusted POS GAMs.

Predictor Variable	Self-Reported Leisure-Time Brisk Walking			Objectively-Measured-MVPA		
	β	95% CI	*p*	β	95% CI	*p*
Coast line contained or intersected by buffer (km)	−59.806	−171.074–51.46	0.293	−32.999	−127.145–61.146	0.493
Healthy routes contained or intersected by buffer (km)	1.376	−1.798–4.549	0.396	1.491	−1.187–4.169	0.277
No. sports facilities contained or intersected by buffer	1.692	−3.079–6.462	0.488	1.580	−2.449–5.609	0.443
Area of sports facilities contained or intersected by buffer (Km^2)	207.995	−113.202–529.19	0.206	−72.160	−344.378–200.058	0.604
No. parks contained or intersected by buffer	−0.267	−2.132–1.597	0.779	0.353	−1.222–1.928	0.661
Areas of parks contained or intersected by buffer (km^2)	−12.612	−29.097–3.872	0.135	−10.196	−24.129–3.737	0.153
No. beaches contained or intersected by buffer	18.841	−7.084–44.766	0.156	−6.006	−27.998–15.987	0.593
Areas of beaches contained or intersected by buffer (km^2)	716.339	−852.14–2284.82	0.372	−324.53	−1651.44–1002.37	0.632
No. POS contained or intersected by buffer	0.063	−1.52–1.646	0.938	0.406	−0.931–1.742	0.552
Areas of POS contained or intersected by buffer (km^2)	−11.960	−28.438–4.517	0.156	−10.403	−24.32–3.514	0.144

Abbreviations: β, non-standardized coefficient; 95% CI, confidence interval; *p*, *p*-value; MVPA, moderate-to-vigorous physical activity in bouts of at least 10 min. β indicates change in self-reported leisure-time brisk walking or OM-MVPA per minutes per day (min/day) per increment (in 1 km, 1 km^2, or count) in access to public open spaces (POS). Individual-level covariate (sex, education level and self-rated health).

Supplementary Table S3. Summary of the results associations between objectively-access to Public Open Spaces (POS) for 500 m sausage network walkable street buffer and 10 minutes bouts OB-MVPA. The results for the following model comparisons are provided: Covariate-adjusted POS GAMMs.

Predictor Variable	[a] Objectively-Assessed MVPA 10 Minutes Bouts (Minutes/Day) (n = 216)			[b] Engaging in ≥150 min/week Objectively-Assessed MVPA 10 min Bouts (n = 216)		
	β	95% CI	*p*	OR	95% CI	*p*
Healthy routes contained or intersected by buffer (km)	1.334	−1.357–4.025	0.332	1.083	0.81–1.448	0.592
No. sports facilities contained or intersected by buffer	1.078	−3.094–5.249	0.613	1.234	0.813–1.874	0.325
No. parks contained or intersected by buffer	0.522	−1.159–2.203	0.544	1.058	0.894–1.25	0.518
Areas of parks contained or intersected by buffer (km^2)	−7.474	−20.942–5.994	0.278	0.304	0.039–2.335	0.253
No. POS contained or intersected by buffer	0.459	−0.971–1.89	0.53	1.061	0.922–1.22	0.411
Areas of POS contained or intersected by buffer (km^2)	−7.697	−21.161–5.766	0.264	0.308	0.041–2.319	0.254

Abbreviations: β, non-standardized coefficient; OR, odds ratio; 95% CI, confidence interval; *p*, *p*-value. β, indicates change in 10 min bouts OB-MVPA per minutes per day (min/day) per increment (in 1 km, 1 km^2 or count) in access to public open spaces (POS). All coefficients are adjusted for individual-level covariate (sex, age, education level and self-rated health). [a] GAMMs with gaussian variance and identity link functions. [b] GAMM with binomial variance and logit link functions.

Supplementary Table S4. Summary of the interaction between objectively-access to Public Open Spaces (POS) for distance and 1000m sausage network walkable street buffer and 10 minutes bouts OB-MVPA. The results for the following model comparisons are provided: Covariate-adjusted POS GAMMs.

Predictor Variable	[a] Objectively-Assessed MVPA 10 min Bouts (Minutes/Day) (*n* = 216)				[b] Engaging in ≥ 150 min/week Objectively-Assessed MVPA 10 minBouts (*n* = 216)			
	p. int	β	95% CI	*p*	*p*.int	OR	95% CI	*p*
Distance to the coast (km)	0.403	0.794	−1.733–3.32	0.539	0.253	1.148	0.906–1.454	0.255
Distance to the healthy route for walking (km)	0.412	−1.422	−8.894–6.049	0.709	0.085	0.871	0.478–1.588	0.653
Distance to the nearest sports facility (km)	0.230	−3.626	−10.019–2.767	0.268	0.333	0.861	0.47–1.576	0.627
Distance to the nearest beach (km)	0.996	−1.102	−3.929–1.726	0.446	0.448	1.000	0.769–1.302	0.997
Distance to the nearest Park (km)	0.832	−4.110	−14.316–6.097	0.431	0.480	0.770	0.317–1.869	0.565
Healthy routes contained or intersected by buffer (km)	0.028	1.912	0.622–3.202	0.004	0.071	1.120	0.978–1.282	0.103
No. sports facilities contained or intersected by buffer	0.156	2.290	−0.575–5.155	0.119	0.599	1.103	0.83–1.464	0.501
No. parks contained or intersected by buffer	0.894	0.503	−0.305–1.312	0.224	0.532	1.000	0.922–1.084	0.994
Areas of parks contained or intersected by buffer (km^2)	0.939	−4.568	−19.597–10.46	0.552	0.874	0.743	0.153–3.618	0.713
No. POS contained or intersected by buffer	0.655	0.487	−0.201–1.174	0.167	0.678	1.005	0.939–1.076	0.882
Areas of POS contained or intersected by buffer (km^2)	0.945	−4.372	−19.398–10.654	0.569	0.974	0.731	0.15–3.576	0.699

Abbreviations: *p*.int, *p*-value for interaction each POS and rain during the accelerometer wearing period; β, non-standardized coefficient OR, odds ratio; 95% CI, confidence interval; *p*, *p*-value. β, indicates change in 10 minutes bouts OB-MVPA per minutes per day (min/day) per increment (in 1 km, 1 km^2 or count) in access to public open spaces (POS). All coefficients are adjusted for individual-level covariate (sex, age, education level and self-rated health). [a] GAMMs with gaussian variance and identity link functions. [b] GAMM with binomial variance and logit link functions.

Supplementary Table S5. Summary of the interaction between objectively-access to Public Open Spaces (POS) for 500 m sausage network walkable street buffer and 10 minutes bouts OB-MVPA. The results for the following model comparisons are provided: Covariate-adjusted POS GAMMs.

Predictor Variable	[a] Minutes/day Objectively-Assessed MVPA 10 min Bouts (n = 216)				[a] Engaging in ≥150 min/week Objectively-Assessed MVPA 10 min Bouts (n = 216)			
	***p*. int**	**β**	**95% CI**	***p***	***p*.int**	**OR**	**95% CI**	***p***
Healthy routes contained or intersected by buffer (km)	0.135	3.410	–0.395–7.215	0.081	0.175	1.303	0.879–1.933	0.188
No. sports facilities contained or intersected by buffer	0.471	2.458	–3.03–7.947	0.381	0.731	1.307	0.765–2.233	0.329
No. parks contained or intersected by buffer	0.455	1.008	–1.191–3.207	0.370	0.299	1.134	0.913–1.409	0.256
Areas of parks contained or intersected by buffer (km^2)	0.640	–4.915	–22.461–12.63	0.584	0.817	0.368	0.03–4.502	0.435
No. POS contained or intersected by buffer	0.373	0.939	–0.879–2.757	0.312	0.316	1.119	0.937–1.336	0.215
Areas of POS contained or intersected by buffer (km^2)	0.628	–5.047	–22.597–12.503	0.574	0.791	0.382	0.033–4.495	0.445

Abbreviations: *p*.int, *p*-value for interaction each POS and rain during the accelerometer wearing period; β, non-standardized coefficient OR, odds ratio; 95%CI, confidence interval; *p*, *p*-value. β, indicates change in 10 min bouts OB-MVPA per minutes per day (min/day) per increment (in 1 km, 1 km^2 or count) in access to public open spaces (POS). All coefficients are adjusted for individual-level covariate (sex, age, education level and self-rated health). [a] GAMMs with gaussian variance and identity link functions. [b] GAMM with binomial variance and logit link functions.
